# GluN2A- and GluN2B-containing pre-synaptic *N*-methyl-d-aspartate receptors differentially regulate action potential-evoked Ca^2+^ influx via modulation of SK channels

**DOI:** 10.1098/rstb.2023.0222

**Published:** 2024-07-29

**Authors:** Carla C. Schmidt, Rudi Tong, Nigel J. Emptage

**Affiliations:** ^1^ Department of Pharmacology, University of Oxford, Oxford OX1 3QT, UK; ^2^ Montreal Neurological Institute, 3801 University Street, Montreal, Quebec H3A 2B4, Canada

**Keywords:** NMDA receptor, short-term plasticity, homeostatic plasticity, SK channel, pre-synaptic terminal

## Abstract

*N*-methyl-d-aspartate receptors (NMDARs) play a pivotal role in synaptic plasticity. While the functional role of post-synaptic NMDARs is well established, pre-synaptic NMDAR (pre-NMDAR) function is largely unexplored. Different pre-NMDAR subunit populations are documented at synapses, suggesting that subunit composition influences neuronal transmission. Here, we used electrophysiological recordings at Schaffer collateral-CA1 synapses partnered with Ca^2+^ imaging and glutamate uncaging at boutons of CA3 pyramidal neurones to reveal two populations of pre-NMDARs that contain either the GluN2A or GluN2B subunit. Activation of the GluN2B population decreases action potential-evoked Ca^2+^ influx via modulation of small-conductance Ca^2+^-activated K+ channels, while activation of the GluN2A population does the opposite. Critically, the level of functional expression of the subunits is subject to homeostatic regulation, bidirectionally affecting short-term facilitation, thus providing a capacity for a fine adjustment of information transfer.

This article is part of a discussion meeting issue ‘Long-term potentiation: 50 years on’.

1. Introduction

The *N*-methyl-d-aspartate receptor (NMDAR) is well established as playing a critical role in synaptic plasticity and neural computation [1–3] with its function extensively explored at the post-synaptic terminal [4–8]. The presence of NMDARs at pre-synaptic terminals is well documented across a variety of brain areas, with several functional roles ascribed to them [9–22]. However, their elusiveness towards experimental interrogation and detection has hampered significant progress in understanding their role as modulators of synaptic transmission [12].

NMDARs are diheteromers comprising two GluN1 subunits and two GluN2 (GluN2A, GluN2B, GluN2C and GluN2D) or GluN3 (GluN3A and GluN3B) subunits [23,24] with GluN2A and GluN2B being the predominant subunits found in the mammalian forebrain [23,25,26]. NMDAR subunit composition creates functional diversity through subunit-specific differences in ion permeability, channel gating and conductance and coupling to accessory regulatory proteins [24,27–29]. At the post-synaptic terminal, NMDAR subunit composition has been linked to distinct processing pathways, often serving opposing roles. For example, GluN2 subunits within hippocampal neurones selectively mediate the direction of plasticity through the regulation of Ca^2+^ influx [30,31]. The inhibition of GluN2B-containing NMDARs prevents long-term depression (LTD), whereas blockade of GluN2A-containing NMDARs inhibits long-term potentiation (LTP) [26,32–35]. Additionally, NMDAR subunit composition plays a pivotal role in shaping the temporal dynamics of synaptic responses [21,36–38].

Far less well explored is the differential role of NMDAR subunit composition at the pre-synaptic terminal, though its importance in various forms of short- and long-term synaptic plasticity is established [9,18,19,39–41]. Previous efforts to investigate the effects of pre-synaptic NMDAR (pre-NMDAR) subunit compositions suggest that pre-NMDARs containing the GluN2B or GluN2C/D subunits at hippocampal CA3–CA1 synapses can enhance glutamate release [42], though the mechanistic details are unclear. Similarly, in cortical neurones, the GluN2B subunit exhibits a tonic facilitatory effect on spontaneous glutamate release [43].

In this study, we identified two distinct pre-NMDAR populations at the pre-synaptic terminal of hippocampal CA3 neurones that contain either GluN2A or GluN2B subunits. The acute activation of these pre-NMDARs either increased (GluN2A) or decreased (GluN2B) the Ca^2+^ influx upon arrival of an action potential (AP) at the bouton. The modulation in Ca^2+^ dynamics required small-conductance Ca^2+^-activated K^+^ channels (SK channels). Furthermore, we show that the composition of pre-NMDAR is sensitive to global network activity and shifts from being GluN2B to GluN2A-dominant as network activity increases. Finally, we show that the pre-NMDAR subunit composition impacts on short-term facilitation and sets the pre-synaptic integration time window and therefore the bandwidth of information transfer.

## 2. Material and methods

### (a) Organotypic hippocampal slices

Transverse 350 μm organotypic hippocampal slices were prepared from male Wistar rats (P7–P8). After dissection, slices were cultured on Millicell CM membranes (polytetrafluoroethylene filter; pore size, 0.4 μm; diameter, 12 mm) and maintained in culture media at 37°C for 7–14 days prior to use. Culture media comprised 78.8% minimum essential media, 20% heat-inactivated horse serum, 30 mM HEPES, 26 mM D-Glu, 5.8 mM NaHCO_3_, 1 mM CaCl_2_, 2 mM MgSO_4_ and 1% B-27. The media was replaced every 2–3 days to ensure optimal and constant conditions for the slices. During the experiments, slices were superfused with oxygenated (95% O_2_/5% CO_2_) artificial cerebrospinal fluid (ACSF; composition in mM: 145 NaCl, 2.5 KCl, 2 MgCl_2_, 3 CaCl_2_, 1.2 NaH2PO_4_, 16 NaH2CO_3_ and 11 glucose). Each slice was used only for a single experiment owing to extensive pharmacological manipulations.

(b) Pharmacology

In total, 200 nM NBQX was added to the ACSF to prevent hyperactivity in organotypic slices. In order to block pre-NMDARs, 50 μM AP5 was either washed in or included in the ACSF from the start. Ro 25-6981 (1 µM), PEAQX (100 nM) and DQP 1105 (50 μM) were used to specifically block NMDAR subunits. For SK-channel inhibition, slices were pre-incubated in 1 µM apamin, which was maintained throughout the experiment. During electrophysiology experiments, slices were perfused with Ro 25-6981 (1 µM) for at least 10 min to ensure reliable blocking of GluN2B. To manipulate global network activity and to induce homeostatic plasticity, slices were incubated for 48–72 h with either gabazine (1 µM) or NBQX (10 µM)/AP5 (50 µM), before imaging.

### (c) Electrophysiology and imaging

Electrophysiological data were recorded in whole-cell patch clamp mode with WinWCP (Strathclyde Electrophysiology 851 software) and analysed using Clampfit (Axon Instruments). Cells were imaged with a LEICA DMLFSA microscope fitted with a 63× water-immersion objective (HCX APO L 63×/0.9W U-V-I; Leica) and a LEICA TCS SP2 confocal scan head. For Ca^2+^ imaging, superficial CA3 pyramidal cells were patched with low-resistance patch electrodes (4–8 MΩ) containing the Ca^2+^-sensitive dye Oregon green 488 BAPTA-1 (OGB-1; 0.5–1 mM) for 1–5 min. We waited at least 30–45 min for the indicator to reach diffusional equilibrium in the axon. We closely monitored signal intensity, and we did not see an increase in the basal fluorescence intensity of the dye over the course of the experiments. Furthermore, dye saturation in the axon was examined by testing the summation of Ca^2+^ responses of two APs given in quick succession. After successful identification of the axon and superficial boutons, cells were repatched. Subsequently, line scans through boutons were performed and synchronized to intrasomatically stimulated APs triggered by injecting step currents (0.5–2 pA) of 10 ms duration. We recorded 10–15 successive trials per condition. Ca^2+^ responses were averaged within trials, and the peak response was extracted. Ca^2+^ transients in boutons were analysed using ImageJ and expressed as the fractional change in fluorescence (∆*F*/*F*):


$$∆F/F=(F_{transient}-F_{baseline})/(F_{baseline}-F_{background}).$$

To elicit high-frequency AP bursts, a monopolar tungsten electrode encased in a glass pipette was positioned in stratum radiatum to stimulate Schaffer collaterals. CA1 pyramidal cells were stimulated with five pulses at 50 Hz followed by a single pulse given 100 ms from the end of burst. Patch electrodes contained 1 mM MK-801 to block post-synaptic NMDARs. Experiments were not conducted blind to experimental conditions.

### (d) Glutamate uncaging

Before each experiment, we titrated the intensity of the uncaging laser (405 nm ultraviolet (UV) laser) to detect a robust rise in Ca^2+^ (0.5–1 ∆*F*/*F*) in distal dendritic spines (electronic supplementary material, figure S1). This calibration ensures the release of glutamate at physiologically relevant concentrations and minimizes phototoxicity. After identification of superficial boutons, a glass pipette (4–8 MΩ) filled with 4-methoxy-7-nitroindolinyl (MNI)-glutamate (10 mM) and connected to a picospritzer was placed close to the boutons (within 20 µm just above the surface of the slice) to ensure focal delivery of the MNI-glutamate. Since intrasomatically induced APs take time to reach the pre-synaptic terminals and to release glutamate, we set the glutamate uncaging to occur 0.5–5 ms after the induced AP (electronic supplementary material, figure S1).

### (e) Statistical analysis

ImageJ and GraphPad Prism 7 were used for analysis, graphing and statistical testing. Data were analysed with a two-tailed Mann–Whitney *U*-test, Wilcoxon signed-rank test or Kruskal–Wallis with post hoc Dunn’s test for multiple comparisons. Data are reported as mean ± standard error of the mean (s.e.m.). Significance is denoted as follows: *p < 0.05, **p < 0.01 and ***p < 0.001.

## 3. Results

### (a) Two distinct populations of pre-synaptic NMDARs bidirectionally modulate action potential-evoked Ca^2+^ influx

**Figure 1.**
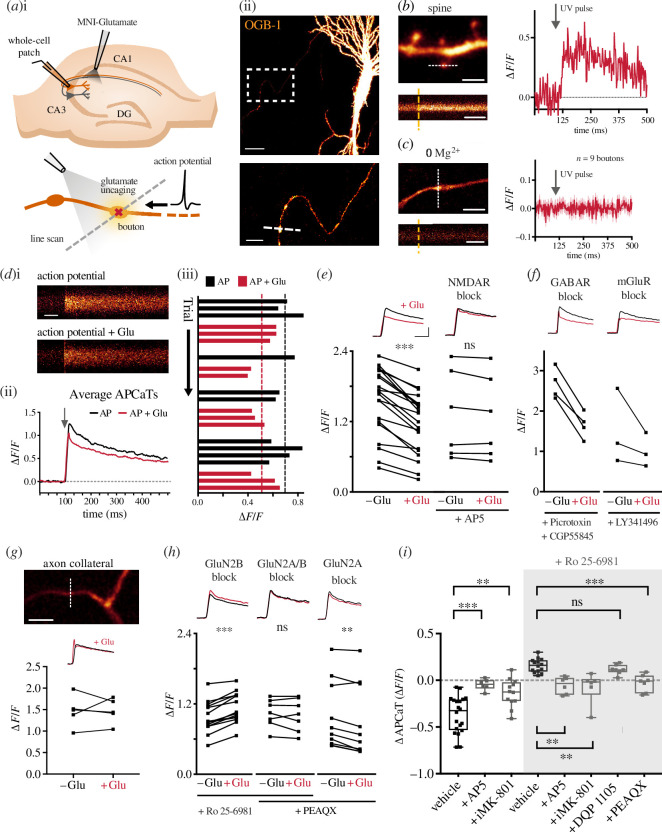
Two distinct populations of pre-NMDARs bidirectionally modulate AP-evoked Ca^2+^ influx. (*a*(i)) Experimental set-up: CA3 cells were patched in whole-cell mode to elicit APs via current injection. A glass pipette attached to a picospritzer enabled delivery of MNI-glutamate to the bouton. A 405 nm laser was used for focal uncaging at single boutons. (*a*(ii)) Top: example CA3 neurone loaded with OGB-1. The white box indicates pre-synaptic boutons along the Schaffer collaterals. Scale bar = 20 µm. Bottom: enlarged image of the axon with several boutons. The dashed white line indicates the line scan through the bouton. Scale bar = 5 µm. (*b*) Left: an example image of a dendrite from a CA3 neurone loaded with OGB-1 (scale bar = 5 µm) and line scan through the spine indicated by the white dotted line. Orange dashed line indicates photolysis pulse (scale bar = 125 ms). Right: the UV uncaging laser was titrated at small spines to elicit a response of approximately 0.5 Δ*F*/*F*. (*c*) Left: example of an axon and bouton (scale bar = 10 µm) and example trace of a line scan through the bouton indicated by the white dotted line. Right: glutamate uncaging in low Mg^2+^ did not elicit a Ca^2+^ response at Schaffer collateral boutons. (*d*(i)) Line scans of APCaTs with or without glutamate photolysis. Scale bar = 50 ms. (*d*(ii)) Average APCaTs. Without glutamate = black, with glutamate = red. (*d*(iii)) Trial-by-trial peak APCaTs. (*e*) Average peak APCaTs in ACSF, *n* = 20 (left) or in the presence of 50 µM AP5, *n* = 6 (right). (*f*) Inhibition of GABA receptors, *n* = 4, or mGluRs, *n* = 3, with picrotoxin (30 µM), CGP5845 (5 µM) and LY341495 (100 µM) did not block the decrease in APCaTs. (*g*) Photolysis experiments were performed at axon collaterals instead of boutons, *n* = 5. Scale bar = 10 µm. No change in APCaTs could be detected following glutamate photolysis at the collateral (bouton versus collateral *p* = 0.004; Mann–Whitney *U*-test). (*h*) Average peak APCaTs in 1 µM Ro 25-6981, *n* = 14 (left), 1 µM Ro 25-6981 with 100 nM PEAQX, *n* = 7 (centre), or 100 nM PEAQX alone, *n* = 10 (right), to block specific pre-NMDAR subunits. GluN2B block diminished the decrease in APCaTs and unmasked an increase in APCaTs. PEAQX blocked the increase in APCaTs. PEAQX alone did not affect the decrease in APCaTs. (*i*) The difference in the peak amplitude between trials with and without glutamate (∆APCaT) is shown for control experiments, experiments with 50 µM AP5 or 1 mM intracellular MK-801 (‘iMK-801’). Experiments in which GluN2B was blocked with Ro 25-6981 are highlighted in the grey box (AP5 versus vehicle *p* < 0.001, *n* = 6, MK-801 versus vehicle *p* = 0.008, *n* = 12, AP5 versus vehicle_Ro_ *p* < 0.003, *n* = 6, MK-801 versus vehicle_Ro_ *p* = 0.005, *n* = 6, PEAQX versus vehicle_Ro_ *p* = 0.006, *n* = 7, DQP 1105 versus vehicle_Ro_ *p* > 0.99, *n* = 7; Kruskal–Wallis with *post hoc* Dunn’s test).

In order to measure the activation of pre-NMDARs, we bolus-loaded CA3 pyramidal neurones in hippocampal slices with the Ca^2+^ indicator OGB-1, identified their axonal arbours and located superficial pre-synaptic terminals as visually distinct varicosities (figure 1*a*; also see §2). We then locally perfused MNI-glutamate to photolytically release glutamate at targeted boutons. To prevent the activation of post-synaptic NMDARs, we blocked the activation of AMPA receptors (10 µM NBQX), which are required for the relief of the Mg^2+^ block at the post-synaptic terminal. The concentration of glutamate was titrated prior to each experiment at spines located at a similar depth to match the Ca^2+^ transients observed during endogenous evoked release of glutamate (figure 1*b*; also see §2). Consistent with the study of Carter and Jahr [44] in cortical neurones, glutamate photolysis did not cause a significant rise in Ca^2+^, even in conditions of low Mg^2+^ (figure 1*c*). We therefore considered whether Ca^2+^ entry may require additional membrane depolarization. To explore this, we paired glutamate photolysis with an AP evoked by current injection via a patch pipette into the neurone under study. To our surprise, pairing an AP with glutamate release between 0.5 and 5 ms following the onset of the AP resulted in a decrease in AP-evoked Ca^2+^ transient (APCaT, *n* = 20 boutons/8 animals, ∆APCaT = −0.347 ± 0.049 ∆*F*/*F*; figure 1*d,e*). This decrease was consistent over multiple trials (figure 1*d*(iii)) but was completely abolished by bath application of AP5 (50 µM; *n* = 6 boutons/3 animals, ∆APCaT = −0.047 ± 0.024 ∆*F*/*F*; figure 1*e*). This suggested the involvement of pre-NMDARs since the activation of post-synaptic NMDARs was probably prevented by the global inhibition of AMPA receptors. Inhibition of metabotropic glutamate receptors (mGluRs) or GABA receptors did not affect the reduction in AP-evoked Ca^2+^ influx (figure 1*f*). We also did not observe a decrease in APCaTs in the axon collaterals (figure 1*g*). We further confirmed the pre-synaptic nature of NMDAR activation by loading the cell with MK-801 (‘iMK-801’, 1 mM) through the patch pipette to specifically block pre-NMDARs within the cell under investigation. This also significantly blocked the decrease in APCaTs (*n* = 12 boutons/5 animals, ∆APCaT = −0.137 ± 0.043 ∆*F*/*F*; figure 1*i*).

The lack of direct Ca^2+^ entry following glutamate photolysis and the glutamate-induced decrease in APCaTs are outcomes quite different from those seen for post-synaptic NMDARs. We hypothesized that this may reflect differences in subunit composition. We repeated the experiment with subunit-specific NMDAR antagonists. Bath application of the GluN2B subunit antagonist Ro 25-6981 (1 µM) led to an increase in APCaTs (*n* = 12 boutons/4 animals, ∆APCaT = 0.157 ± 0.02 ∆*F*/*F*; figure 1*h,i*). This increase was prevented by additionally bath application of either AP5 (50 µM) or intracellular MK-801 (1 mM; figure 1*i*). This indicates that the decrease in APCaTs was mediated by GluN2B-containing pre-NMDARs and, furthermore, that there exists a second population of pre-NMDARs that do not contain the GluN2B subunit mediating an increase in APCaTs. Incidentally, GluN2B inhibition unmasked a small but significant increase in pre-synaptic Ca^2+^ following glutamate photolysis in low Mg^2+^ (electronic supplementary material, figure S1). Next, with GluN2B subunits blocked, we inhibited the GluN2A subunit using PEAQX (100 nM). This completely abolished the increase in APCaTs (*n* = 7 boutons/3 animals, ∆APCaT = −0.029 ± 0.034 ∆*F*/*F*; figure 1*h,i*). Inhibition of the GluN2C/D subunits using DQP 1105 (50 µM) had no effect (*n* = 7 boutons/3 animals, ∆APCaT = 0.116 ± 0.02 ∆*F*/*F*; figure 1*i*). PEAQX application alone did not result in a further decrease in APCaTs (figure 1*h*) and is most probably owing to the lower specificity and partial inhibition of the GluN2B subunit [45,46].

These observations suggest that two populations of NMDARs are present at CA3 pre-synaptic terminals, one that contains the GluN2A subunit and the other the GluN2B subunit. GluN2A-containing pre-NMDARs increase the Ca^2+^ influx following the AP, whereas GluN2B-containing pre-NMDARs decrease it.

### (b) Pre-synaptic NMDARs modulate action potential-evoked Ca^2+^ influx by the activation of SK channels

We next explored the mechanism by which pre-NMDARs modulate AP-evoked Ca^2+^ influx. At the post-synaptic terminal, SK channels are known to form a Ca^2+^-mediated negative feedback loop with NMDARs to reduce Ca^2+^ influx into synaptic terminals [47–49]. Moreover, SK channels are well-established as shaping the AP waveform [50–54], impacting the dynamics of pre-synaptic voltage-gated Ca^2+^ channels (VGCCs). We hypothesized that the NMDAR/SK-channel pathway is also present in CA3 pre-synaptic terminals, where it is able to modulate AP-evoked Ca^2+^ influx.

**Figure 2.**
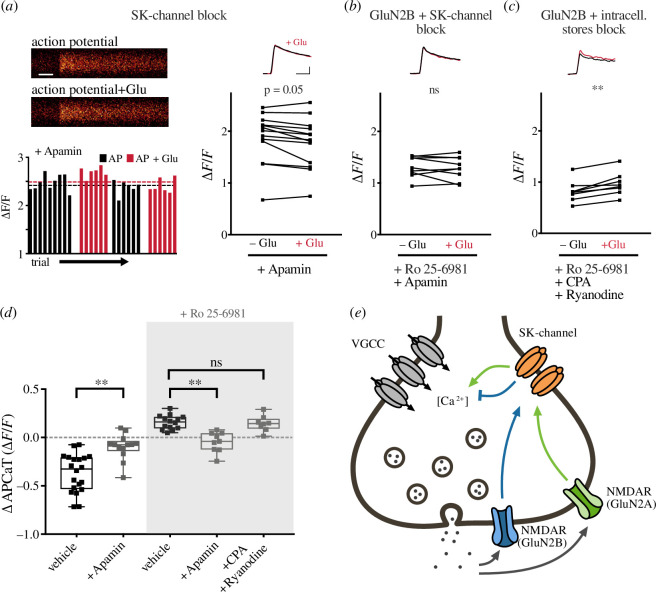
Pre-NMDARs modulate AP-evoked Ca^2+^ influx via the activation of SK channels. (*a*) Top left: line scans of APCaTs following SK-channel inhibition with 1 μM apamin. Scale bar = 50 ms. Bottom left: trial-by-trial peak APCaTs. Without glutamate = black, with glutamate = red. Right: average peak APCaTs in 1 µM apamin. The decrease in peak APCaTs was diminished after SK-channel inhibition, *n* = 13. (*b,c*) Similarly, the Ro 25-6981-dependent increase in peak APCaTs was abolished after application of 1 µM apamin, *n* = 9. Inhibition of intracellular Ca^2+^ stores using CPA (15 µM) and ryanodine (20 µM) did not affect the increase, *n* = 8. (*d*) The difference in the peak amplitude of APCaTs is shown for experiments in ACSF, *n* = 20 (from figure 1) and experiments with 1 µM apamin. Experiments in which GluN2B was blocked with Ro 25-6981 are highlighted in the grey box (apamin versus vehicle *p* < 0.001, apamin versus vehicle_Ro_ *p* = 0.001, CPA and ryanodine versus vehicle_Ro_ *p* > 0.99; Kruskal–Wallis with *post hoc* Dunn’s test). Error bars represent s.e.m. (*e*) Proposed molecular mechanism for pre-NMDAR and SK-channel-mediated boutonal Ca^2+^ dynamics. The release of glutamate activates two different pre-NMDAR populations, containing either GluN2A or GluN2B, which causes local Ca^2+^ influx and leads to the modulation of SK channels, resulting in an increase or a decrease in Ca^2+^ influx, respectively, probably through VGCCs.

Bath application of the selective SK-channel blocker apamin (1 µM) abolished both the glutamate photolysis-induced decrease (*n* = 13 boutons/4 animals, ∆APCaT = −0.097 ± 0.037 ∆*F*/*F*; figure 2*a,d*) and increase in APCaTs (*n* = 9 boutons/3 animals, ∆APCaT = −0.046 ± 0.035 ∆*F*/*F*; figure 2*b,d*), observed following the application of Ro 25-6981. This suggests that both GluN2A- and GluN2B-containing pre-NMDARs act via SK channels to modulate pre-synaptic Ca^2+^ dynamics. We also thought it important to assess whether intracellular stores formed part of the pathway and so applied cyclopiazonic acid (CPA, 15 µM) and ryanodine (20 µM); however, these did not affect the increase in APCaTs (*n* = 8 boutons/3 animals, ∆APCaT = 0.144 ± 0.029 ∆*F*/*F*; figure 2*c,d*).

We conclude from these observations that following the release of glutamate, two populations of pre-NMDARs become activated, each triggering a cascade of intracellular signalling events that converge upon SK channels (figure 2*e*). The SK channels influence the duration of the AP and, consequently, the opening of VGCCs. Whether an increase or a decrease in the AP-evoked Ca^2+^ influx occurs reflects the dominance of one pathway over the other.

### (c) Network activity shifts the functional balance between the GluN2A and GluN2B sub-populations

**Figure 3.**
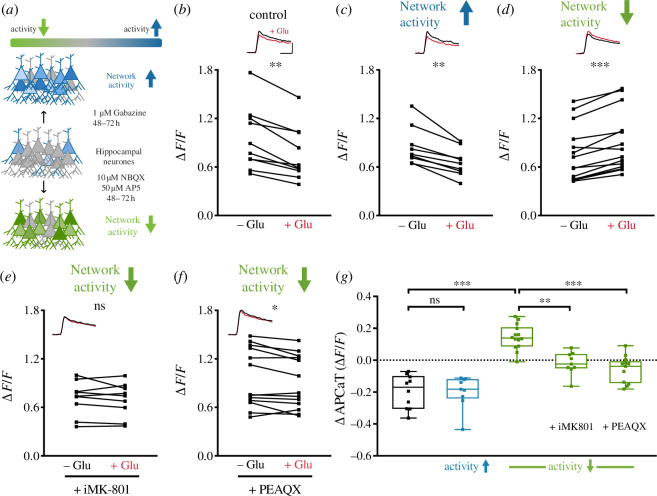
Network activity shifts the balance between GluN2A and GluN2B sub-populations. (*a*) Schematic representation of the experimental conditions. Network activity was either increased or decreased via application of 1 µM gabazine or 10 µM NBQX and 50 µM AP5 for 48–72 h, respectively. (*b–d*) Peak APCaTs in control conditions, *n* = 10 (*b*) and after increasing, *n* = 9 (*c*) or decreasing, *n* = 14 (*d*) network activity. Decreasing network activity led to an increase in peak APCaTs (*d*). This increase was abolished with intracellular MK-801, *n* = 9 and PEAQX, *n* = 13 (*e,f*). (*g*) Summary of the regulation of sub-population balance by network activity (vehicle_lowactivity_ versus control *p* < 0.001, vehicle_lowactivity_ versus iMK-801 *p* = 0.002, PEAQX versus vehicle_lowactivity_ *p* < 0.001; Kruskal–Wallis with *post hoc* Dunn’s test). Error bars represent s.e.m.

What determines the dominant pre-NMDAR sub-population at a given synapse? We reasoned that the composition of pre-NMDARs might be able to modulate pre-synaptic neurotransmitter release given its strong dependence on the dynamics of Ca^2+^ influx. We hypothesized that the balance between sub-populations may be modified by global network activity, perhaps in a homeostatic manner. We tested this by globally increasing (1 µM gabazine) or decreasing (10 µM NBQX and 50 µM AP5) network activity for 48–72 h (figure 3*a*), an established protocol for induction of homeostatic plasticity [55]. We then measured the modulation of APCaTs.

Increased network activity resulted in an overall decrease in APCaTs (*n* = 9 boutons/4 animals, ∆APCaT = −0.2 ± 0.034 ∆*F*/*F*; figure 3*c*), which was not significantly different from control experiments (gabazine versus CTR *p* > 0.99; Kruskal–Wallis with *post hoc* Dunn’s test; figure 3*b,c,g*). By contrast, decreasing network activity resulted in a substantial increase in APCaTs (*n* = 14 boutons/4 animals, ∆APCaT = 0.144 ± 0.021 ∆*F*/*F*; figure 3*d,g*) following photolysis of glutamate. We verified that this increase is caused by an increase in GluN2A-containing pre-NMDARs via intracellular MK-801 (*n* = 9 boutons/4 animals, ∆APCaT = −0.019 ± 0.024 ∆*F*/*F*; figure 3*e,g*) and application of PEAQX (*n* = 13 boutons/4 animals, ∆APCaT = −0.057 ± 0.023 ∆*F*/*F*; figure 3*f,g*). Application of PEAQX did not unmask a decrease in APCaTs (figure 3*g*), suggesting that the extensive silencing of network activity has caused a shift in the dominant sub-population expression such that far fewer GluN2B-containing receptors remained.

### (d) Pre-synaptic NMDAR modulation of action potential-evoked Ca^2+^ influx results in use-dependent modulation of short-term facilitation

Does the change in Ca^2+^ influx modulate neurotransmitter release? Changes in pre-synaptic Ca^2+^ signalling predominantly impact neurotransmitter release and short-term plasticity (STP). In particular, the accumulation of intracellular Ca^2+^ within the bouton following APs is largely associated with short-term facilitation, a transient increase in release probability [56]. Since pre-NMDARs modulate the amount of Ca^2+^ entering the bouton, we hypothesized that pre-NMDARs act to modify short-term facilitation.

**Figure 4.**
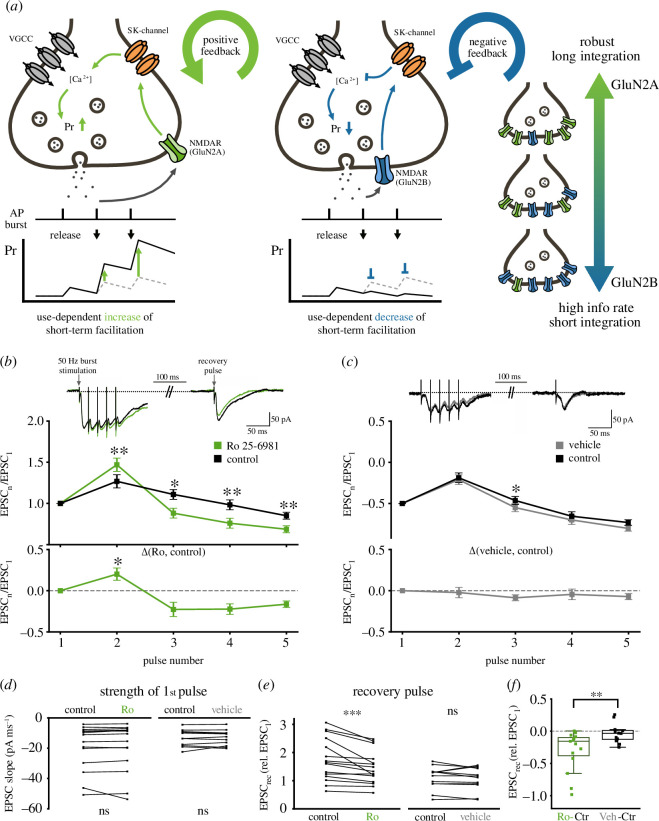
Pre-NMDAR modulation of AP-evoked Ca^2+^ influx results in use-dependent modulation of short-term facilitation. (*a*) Potential consequence of use-dependent modulation of short-term facilitation. The activation of the GluN2A dominant pathway leads to a use-dependent increase in short-term facilitation, which ensures that multiple quanta of release will be transmitted by a given AP burst. This results in a temporal extension of the AP burst, which increases the integration time window at the post-synaptic neurone. The GluN2B-dominant pathway, following glutamate release, acts to prevent the increase in *P*_*r*_ caused by short-term facilitation. This use-dependent ‘clamp’ of short-term facilitation prevents excessive vesicle depletion (i.e. short-term depression) and resets the synapse for subsequent AP trains, allowing for fast information transfer with short integration time windows. (*b*) Top: sample traces of burst stimulation (five APs at 50 Hz) before (black) and after the addition of 1 μM Ro 25-6981 (green) to block GluN2B-containing pre-NMDARs. Middle: average excitatory post-synaptic current (EPSC) normalized to baseline during burst stimulation before and after the addition of Ro 25-6981. Application of Ro 25-6981 significantly increased the magnitude of short-term facilitation (*n* = 15 cells; *p* = 0.001 for pulse 2; Wilcoxon signed-rank test) and enhanced the magnitude of short-term depression (*n* = 15 cells; *p* < 0.05 for pulse 3–5; Wilcoxon signed-rank test). Bottom: the difference in the EPSC of pulses 1–5 before and after the addition of Ro 25-6981 (significance indicates test against vehicle in *c*; *p* < 0.05 for pulse 2; Mann–Whitney test). (*c*) Same as (*b*) for vehicle (ACSF alone, *n* = 11 cells). (*d*) The initial EPSC does not change during the experiment (*n* = 15 cells (control), *p* = 0.08; *n* = 11 cells (vehicle), *p* > 0.99; Wilcoxon signed-rank test). (*e,f*) The magnitude of the recovery pulse given 100 ms after the burst was significantly decreased when GluN2B activity was inhibited with Ro 25-6981 but not in control experiments (Ro 25-6981: *p* < 0.001; vehicle: *p* = 0.34; Wilcoxon signed-rank test; Ro versus vehicle: *p* < 0.01, Mann–Whitney test).

The requirement of glutamate for the activation of the pre-NMDARs adds an additional use-dependent component to short-term facilitation, i.e. it is not solely influenced by the clearance rate of Ca^2+^. Figure 4*a* illustrates the potential outcomes of use-dependent modulation of short-term facilitation. The activation of the GluN2A dominant pathway forms a positive feedback loop in which the release of glutamate will augment neurotransmitter release during a train of APs. This mechanism ensures that multiple release events occur for an AP burst, increasing the robustness of information transmission, by extending the impact of the burst in time, thereby increasing the integration time window at the post-synaptic neurone (figure 4*a*, left). By contrast, the GluN2B-dominant pathway forms a negative feedback loop that reduces the increase in *P*_*r*_ during bursts of APs. For this pathway to be active, neurotransmitter release must of course first occur, a condition that ensures that some neurotransmitter is always released. This use-dependent ‘clamping’ of short-term facilitation prevents excessive vesicle depletion (i.e. short-term depression) and resets the synapse for subsequent AP trains (figure 4*a*, middle). This will enhance information transfer within short integration time windows. The precise balance between GluN2A and GluN2B sub-populations can therefore set the optimal transmission mode for a given synapse.

We tested whether pre-NMDAR subunit populations differentially regulate short-term facilitation. To do so, we recorded from CA1 neurones in response to bursts of APs (five pulses at 50 Hz) elicited at the Schaffer collateral inputs. We also included a single recovery pulse 100 ms after the burst (figure 4*b*, top) to examine the speed of recovery from short-term depression. In total, 1 mM MK-801 was included in the patch electrode to block post-synaptic NMDARs.

AP trains elicited short-term facilitation, followed by a slow decline (figure 4*b,c*) as previously reported for these synapses [57–61]. As we have previously shown, the GluN2B pathways are dominant in the basal state of the network. Application of the GluN2B subunit blocker Ro 25-6981 (1 µM) significantly increased short-term facilitation of the second pulse and enhanced short-term depression of subsequent pulses (figure 4*b*). STP remained unchanged when ACSF alone was perfused (figure 4*c*). Basal *P*_*r*_ is a key determinant of the short-term behaviour of a synapse. We therefore wished to rule out the possibility that the application of Ro 25-6981 caused a change in *P*_*r*_. For this, we compared the magnitude of the first pulse in each burst (Ro 25-6981: *n* = 15 cells/5 animals, *p* = 0.09; vehicle: *n* = 11 cells/5 animals, *p* > 0.99; Wilcoxon signed-rank test; figure 4*d*). We did not find a significant difference. We next examined the recovery from short-term depression. The magnitude of the single recovery pulse given 100 ms after the burst was significantly decreased when GluN2B activity was inhibited with Ro 25-6981 (Ro 25-6981: *n* = 15 cells/5 animals, *p* < 0.001; vehicle: *n* = 11 cells/5 animals, *p* = 0.34; Wilcoxon signed-rank test; figure 4*e*), but not in control vehicle experiments (Ro 25-6981 versus vehicle: *p* = 0.01; Mann–Whitney *U*-test; figure 4*f*).

Consistent with our hypothesis, the results show that pre-NMDARs can regulate short-term facilitation in a use-dependent manner. This mechanism allows for fine adjustment of the information transfer properties of the terminal.

4. Discussion

Here, we showed that the activation of two pre-NMDAR populations, each with a distinct subunit composition, regulates Ca^2+^ dynamics and STP at Schaffer collateral boutons. Pre-NMDARs containing the GluN2B subunit form a negative feedback loop, via SK channels, which decreases the Ca^2+^ influx that occurs during an AP, leading to a reduction in short-term facilitation during high-frequency firing. Pre-NMDARs containing the GluN2A subunit increase the AP-driven Ca^2+^ influx into the bouton, which results in the reinforcement of short-term facilitation during burst firing.

We show that the GluN2B signalling dominates in conditions of high network activity, as commonly observed in hippocampal slice preparations [62], whereas GluN2A signalling dominates when network activity is low. This may explain why the detection of glutamate-evoked Ca^2+^ influx through pre-NMDARs has proved challenging [63–65] including in our own study (figure 1*c*). However, when GluN2B subunits were blocked, we were able to observe a small but significant increase in Ca^2+^, even in the absence of APs (electronic supplementary material, figure S1). Hence, our data support the idea that synaptic terminals can adjust the balance between GluN2A and GluN2B sub-populations depending on the activity level in the network. Whether this balance can be influenced by local activity, i.e. the level of pre- and post-synaptic activity experienced by an individual synapse, remains to be explored, although this appears possible as synapse-specific differences in the expression of pre-NMDARs within the same neurone have been reported in cortex [13]. This result would also add support to a recently proposed model for pre-synaptic computation [66]. Whether the upregulation of GluN2A-containing pre-NMDARs during phases of low network activity results from local translation [67,68] or membrane trafficking [69–71] also remains to be investigated.

Within the hippocampus, a large variety of NMDAR subunits and isoforms can be found, though the extent to which organotypic slice preparations recapitulate the expression profile of the intact, adult animal is unclear. Furthermore, only male rats were used in this study, and potential sex differences remain to be determined. However, pre-NMDARs have been observed in several immunolabelling studies [72,73], and pre-NMDARs have been implicated in pre-synaptic LTD in acute slice preparations [19] and modulation of axon excitability [74]. Next, a receptor’s functional performance is thought to be primarily determined by the GluN2 subunit [23,24,27,29]. In our study, we focused on GluN2A and GluN2B as they are the predominantly expressed isoforms in the adult hippocampus. By contrast, GluN2C and GluN2D expression levels in the adult brain are considerably lower and most prominent in the cerebellum and the brainstem [23,24,29,37,75] or astrocytes [76]. Although the majority of studies have focused on diheteromeric GluN1/GluN2 receptors, the importance of triheteromeric NMDARs is also recognized [28,77–80], raising the possibility for even broader functional diversity. Here, our observations for the pre-NMDAR sub-population involved in the negative feedback loop (containing GluN2B) are consistent with both a GluN1/GluN2B diheteromer and a GluN1/GluN2A/GluN2B triheteromer and therefore require a more detailed pharmacological and genetic dissection of subunit composition.

It is well established that SK channels are activated by Ca^2+^ influx through NMDARs [81–83], which reduces Ca^2+^ influx through VGCCs [25,48,52]. The negative feedback between NMDARs and SK channels has been carefully studied at the post-synaptic terminal [47–49], but not at the pre-synaptic terminal, even though SK channels and NMDARs are known to be colocalized there [49,84]. In this study, we confirm the presence of the negative feedback interaction at the pre-synaptic terminal and link it specifically to the GluN2B subunit of the NMDAR. Additionally, we have shown that the GluN2A subunit is required for the formation of a positive feedback loop with SK channels. This subunit-dependent bidirectional signalling is analogous to the well-characterized roles of post-synaptic NMDARs in LTP and LTD [30,31,33,34].

How do the two pre-NMDAR populations produce their differential effects on the SK channels? While GluN2A and GluN2B show similar characteristics for Ca^2+^ permeability, sensitivity to Mg^2+^ blockade and channel conductance, GluN2A-containing receptors are coupled to distinct downstream signalling networks [24,29]. SK channels are part of large protein complexes and are co-assembled with protein kinase CK2 and phosphatase PP2A, which have opposing effects on SK-channel activity [85–87]: while CK2 phosphorylates SK-channel-bound calmodulin (CaM) resulting in faster channel deactivation and reduced Ca^2+^ sensitivity, PP2A dephosphorylates CaM causing enhanced Ca^2+^ sensitivity [87]. Therefore, it is likely that activation of GluN2A receptors increases CK2 activity within the bouton, leading to an inhibition of SK channels thus prolonging membrane depolarization and consequently Ca^2+^ influx [48,49], whereas activation of the GluN2B-containing sub-population engages the PP2A signalling pathway, leading to a reduction in the depolarization caused by the AP.

Finally, the differential expression patterns of pre-NMDAR subunits and the resulting functional heterogeneity we have identified may account for some of the inconsistencies in the literature. Functional and anatomical evidence for pre-NMDARs has been available for a number of years [18,88–90]; however, the existence of functional receptors has been challenged as some studies report NMDAR-dependent Ca^2+^ transients in boutons [13,18] while others do not [63–65]. Here, we are only able to detect a pre-NMDAR-dependent modulation of Ca^2+^ when activation was paired with APs. Furthermore, only after isolating the GluN2A subunit is Ca^2+^ influx through the pre-NMDARs unmasked (electronic supplementary material, figure S1). It therefore seems prudent to review the data with careful oversight of pre-NMDAR subunit composition.

## Data Availability

Data generated in the current study and analysis code are available at [91]. Electronic supplementary material is available online at [92].
